# A de novo *ANK1* mutation associated to hereditary spherocytosis: a case report

**DOI:** 10.1186/s12887-019-1436-4

**Published:** 2019-02-18

**Authors:** Ti-Long Huang, Bao-Hua Sang, Qing-Ling Lei, Chun-Yan Song, Yun-Bi Lin, Yu Lv, Chun-Hui Yang, Na Li, Yue-Huang Yang, Xian-Wen Zhang, Xin Tian

**Affiliations:** 1 Department of Hematology, Kunming Children’s Hospital, Kunming, China; 2 Medical Faculty, Kunming University of Science and Technology, No.727 Jingming South Road, Kunming, 650500 China

**Keywords:** Hereditary spherocytosis- *ANK1*- frameshift mutation

## Abstract

**Background:**

Hereditary spherocytosis (HS) is a type of hemolytic anemia caused by abnormal red cell membrane skeletal proteins with few unique clinical manifestations in the neonate and infant. An *ANK1* gene mutation is the most common cause of HS.

**Case presentation:**

The patient was a 11-month-old boy who suffered from anemia and needed a regular transfusion therapy at an interval of 2–3 months. Hematological investigations showed moderate anemia (Hb80 g/L). Red cells displayed microcytosis (MCV76.4 fl, MCH25.6 pg, MCHC335 g/L). The reticulocytes were elevated (4.8%) and the spherocytes were increased (10%). Direct antiglobulin test was negative. Biochemical test indicated a slight elevation of bilirubin, mainly indirect reacting (TBIL32.5 μmol/L, IBIL24 μmol/L). The neonatal HS ratio is 4.38, obviously up the threshold. Meanwhile, a de novo *ANK1* mutation (exon 25:c.2693dupC:p.A899Sfs*11) was identified by next-generation sequencing (NGS). Thus, hereditary spherocytosis was finally diagnosed.

**Conclusions:**

Gene detection should be considered in some hemolytic anemia which is difficult to diagnose by routine means. We identified a novel de novo *ANK1* heterozygous frameshift mutation in a Yi nationality patient while neither of his parents carried this mutation.

**Electronic supplementary material:**

The online version of this article (10.1186/s12887-019-1436-4) contains supplementary material, which is available to authorized users.

## Background

Hereditary spherocytosis (HS) results from defects in erythrocyte membrane proteins characterized by hemolysis, anemia, jaundice, gallstones and splenomegaly [1, 2]. The severity depends on rate of hemolysis, degree of compensation of anemia by reticulocytosis. The clinical manifestations vary widely, ranging from nearly asymptomatic to transfusion-dependent or severe life-threatening anemia. In the neonatal period, the major clinical manifestations are jaundice and anemia. Splenomegaly and spherocytes are rarely observed [3]. Therefore, it’s difficult to diagnose in neonates. Even during the first year of life, approximately 34% affected infants are diagnosed [4].

Previous researches have shown that mutations in *ANK1* (ankyrin 1), *SPTB* (spectrin, beta, erythrocytic), *SPTA1* (spectrin alpha, erythrocytic 1), *SLC4A1* (solute carrier family 4, member 1, or band 3), and *EPB42* (erythrocyte membrane protein band 4.2) are associated with HS [5]. The mutations of these genes lead to the normally double-concave disc-shaped red cells become spherical, fragile red cells [6]. *ANK1* located on 8p11.21, its mutations include nonsense, splicing or frameshift and affect about half of patients with HS [7].

In most cases, HS is usually diagnosed on the basis of a positive family history, increased osmotic fragility, hyperbilirubinemia, reticulocytosis, splenomegaly and spherocytes on peripheral blood smears [8]. The neonatal HS ratio which is calculated by dividing the mean corpuscular hemoglobin concentration (MCHC) by the mean corpuscular volume (MCV) provides valuable information for the physicians. In the index infant, the ratio was > 0.36, which points towards a diagnosis of HS (97% sensitivity, 99% specificity) [3]. However, mild or atypical cases are difficult to identify because of the limitations of the classical approaches. It has been reported that approximately 10% patients of HS may be misdiagnosed due to the lack of the typical sphere-shaped erythrocytes in the peripheral blood [9].

In this report, the next-generation sequencing (NGS) was used to analyze a Chinese family with an infant with unknown causes of hemolysis, and we identified a de novo *ANK1* mutation responsible for HS.

## Case presentation

The patient came from a Chinese family in Yunnan province. He showed anemia and jaundice without other pathological symptoms or signs when he was born. Gallstones were identified by B-ultrasound scanning. The results of blood tests before transfusion were shown in the Table 1 which indicated that the child suffered from neonatal moderate hemolytic anemia and hyperbilirubinemia. He had received two blood transfusions in neonates. Autoimmune antibody tests were negative. The neonatal HS ratio is 3.67, only slightly up the threshold.

**Table 1:**
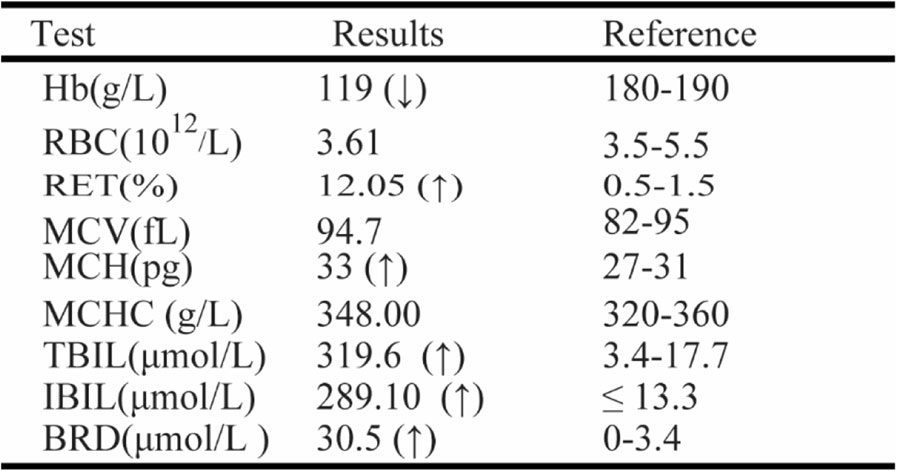
Laboratory test results of the patient at time of birth

*Hb* Hemoglobin, *RBC* red blood cell, *RET* reticulocyte, *MCV* mean corpuscular volume, *MCH* mean corpuscular hemoglobin, *MCHC* mean corpuscular hemoglobin concentration, *TBIL* total bilirubin, *IBIL* indirect bilirubin, *BRD* bilirubin direct

At the age of 3 months, the patient received transfusion because of anemia (Hb: 72 g/L). Before blood transfusion, the following tests were performed. A glucose-6-phosphate dehydrogenase (G-6-PD) screening test and Coombs’ test were negative. Hemoglobin electrophoresis, α and β globin genetic analysis excluded α and β thalassemia. Bone marrow aspiration smears indicated normoblastic hyperplasia. The erythrocyte osmotic fragility wasn’t increased (hemolysis begins: 4.8 g/L referencing 4.4–4.8 g/L; hemolysis completes: 3.2 g/L referencing 2.8–3.2 g/L). Hepatosplenomegaly and spherocytes in peripheral blood smear weren’t observed. The parents were devoid of anemia, jaundice, splenectomy, or early gallstones.

**Fig. 1:**
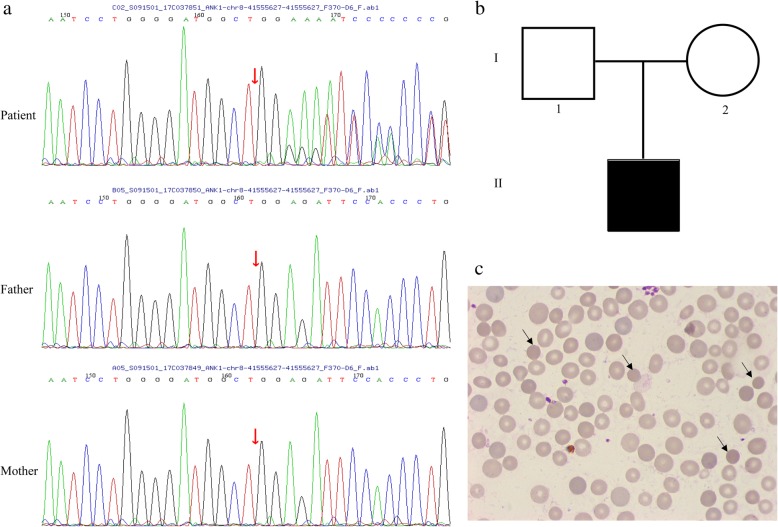
The *ANK1* mutation and pedigree. **a** Sanger sequencing identified an *ANK1* c.2693dupC mutation in the patient. An arrow indicates the mutation site. **b** Family tree and the genotype at the *ANK1* c.2693dupC. Squares and circle denote males and female, respectively. Black symbols denote patient with gene mutation. **c** Peripheral blood smears of the patient. Spherocytes are indicated by arrows

At the age of 6 months, the patient received transfusion again (Hb: 76 g/L). To identify the cause of unexplained hemolysis, we performed genetic analysis by next-generation sequencing according to the methods of He’s [1]. Blood samples were collected before transfusion. Written informed consent for genetic testing was obtained from the parents. DNA was extracted from peripheral blood and 566 genes associated with hematopathy diseases were selected to detect. We detected a mutation in *ANK1* (NM_001142446: exon 25:c.2693dupC: p.A899Sfs*11) in the patient that could be implicated in the patient’s phenotype. The variation resulted in an amino acid change and affected protein function. The mutation was a heterozygous mutation. (Fig. 1a). According to the ESP6500 database, the human genome database and the dbSNP database, this mutation hasn’t been reported previously. However, his parents did not carry this mutation (Fig. 1a-b). Therefore, the patient has a de novo mutation in *ANK1*. In addition, the prevalence of this mutation is extremely low in the population.

At the age of 11 months, pre-transfusion values of the routine blood examination were shown in Table 2 which indicated that he suffered from moderate hemolytic anemia with hyperbilirubinemia. The spherocytes on peripheral blood smear were 10% (Fig. 1c). The neonatal HS ratio is 4.38, obviously up the threshold. He needed a regular transfusion therapy at an interval of 2–3 months with a hemoglobin level of 70–80 g/L before transfusion. The growth and development of the boy are normal. Partial splenic embolization will be planned (Additional file 1).

**Table 2:**
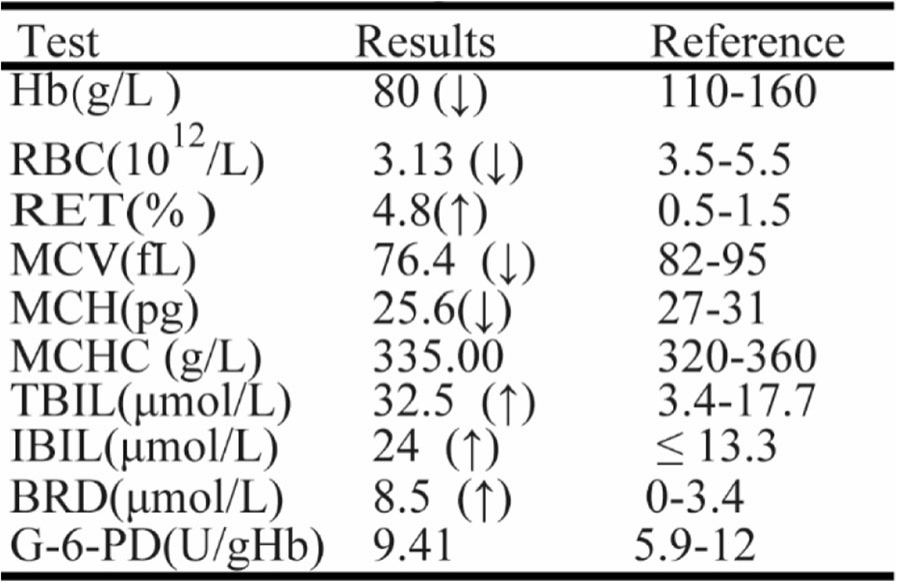
Laboratory test results of the patient at eleven months of age

*Hb* Hemoglobin, *RBC* red blood cell, *RET* reticulocyte, *MCV* mean corpuscular volume, *MCH* mean corpuscular hemoglobin, *MCHC* mean corpuscular hemoglobin concentration, *TBIL* total bilirubin, *IBIL* indirect bilirubin, *BRD* bilirubin direct, *G-6-PD* glucose-6-phosphate dehydrogenase

## Discussion

In this report, we described a Chinese family with a patient affected by HS. A de novo mutation (exon 25: c.2693dupC:p.A899Sfs*11) causing an amino acid change in exon 25 of *ANK1* was found through next-generation sequencing followed by Sanger sequencing to verify the relationship between the *ANK1* mutation and HS.

HS is an inherited disorder characterized by the presence of spherical-shaped blood cells [10, 11]. Approximately two-thirds of cases are autosomal dominant (AD), and the remaining cases represent autosomal recessive (AR) inheritance or de novo mutations in some sporadic cases [12]. Cases of HS are sporadic in China [1]. However, in some countries and continents, many HS patients have no family history [13].

*ANK1* mutations are responsible for the majority of cases of HS. A heterozygous *ANK1* IVS3-2A > C mutation that may lead to exon 4 skipping of the *ANK1* gene and cause HS was recently identified in a 7-year-old girl [14]. A patient with HS who was diagnosed clinically with only 10% spherical-shaped erythrocytes in the peripheral blood was identified to have a novel de novo *ANK1* c.4276C > T (p.R1426*) nonsense mutation, while neither of his parents or his young brother carried this mutation [15]. A 6-year-old girl who was clinically diagnosed with HS carried a de novo nonsense *ANK1* mutation (c.796G > T, p.Glu266X), a single-nucleotide change from G to T, which caused a substitution from glutamic acid to a premature stop at codon 266 [16].

ANK1 is an important red cell membrane protein which plays a vital role in the maintenance of erythrocyte membrane integrity [17, 18]. *ANK1* consists of three structural domains: a multiple repeats N-terminal domain, a spectrin-binding center region and a regulatory C-terminal domain [7, 19, 20]. Mutations in the spectrin-binding domain and regulatory C-terminal domains result in the most severe anemia compared with those located in the other domains [19, 21].

In our study, the patient had suffered from unexplained hemolysis and hyperbilirubinemia since the neonatal period. At the age of 3 months, hepatosplenomegaly and spherocytes which is critical to diagnose the HS weren’t observed [22]. Erythrocyte osmotic fragility was negative. It was difficult to diagnose HS which originates from mutations in the genes coding for RBC membrane components. Gene detection is the principle method for cases with no family history of HS, especially in some atypical cases. NGS is able to provide a thorough genetic analysis and identify which candidate gene is responsible for the disease [23–25]. Therefore, with this patient we used an NGS panel for the analysis of 566 genes responsible for hematological disorders. The genetic tests showed a de novo *ANK1* c.2693dupC (p.A899Sfs*11) frameshift mutation which was not found in the 1000G, ExAC, or HGMD databases. Moreover, this mutation was located in the spectrin-binding domain, which might cause HS. At the age of 11 months, spherocytes on a peripheral blood smear were 10% and the neonatal HS ratio was 4.38. Our report strongly suggests that in infants, it is important for the physicians to monitor the sphere-shaped erythrocytes and the neonatal HS ratio when the patients are at risk for HS. Regrettably, eosin-5′-maleimide binding assay with flow cytometry is the test of choice to diagnose HS but isn’t available in our laboratory.

In conclusion, this report suggests that genetic detecting should be considered for some unexplained hemolytic diseases. Meanwhile, we identified a novel de novo *ANK1* c.2693dupC (p.A899Sfs*11) heterozygous frameshift mutation in a Yi nationality patient. However, the pathogenesis of this *ANK1* mutation should be explored further to improve the diagnosis and treatment of HS.

## Additional file

Additional file 1: Timeline of this case. of a de novo *ANK1* mutation associated to hereditary spherocytosis: a case report. (DOCX 16 kb)

## Abbreviations

AD: Autosomal dominant
AR: Autosomal recessive
EPB42: Erythrocyte membrane protein band 4.2
HS: Hereditary spherocytosis
NGS: Next-generation sequencing
RBC: Red blood cell
SLC4A1: Solute carrier family 4, member 1
SPTA1: Spectrin alpha, erythrocytic 1
SPTB: Spectrin, beta, erythrocytic
