# Supplementary material for: A de novo ANK1 mutation associated to hereditary spherocytosis: a case report
Source: BMC Pediatr. 2019 Feb 18;19:62. doi: 10.1186/s12887-019-1436-4 (PMC6379977; doi:10.1186/s12887-019-1436-4)
Supplement: Supplementary file 1 — Timeline of this case. of a de novo ANK1 mutation associated to hereditary spherocytosis: a case report. (DOCX 16 kb) [file 12887_2019_1436_MOESM1_ESM.docx]

**Timeline (Figure S1)**

The thalassemia gene detection and hemoglobin electrophoresis were normal, Coombs' test is negative.

Peripheral blood smear of 10% spherical red blood cells.

Blood routine: HB72 g/L, blood transfusion therapy

NGS detection found that ANK1 frameshift mutation.

HB 119 g/L. Blood transfusion and

phototherapy were given.

He was pale, yellowish skin; no enlargement of lymph nodes; Liver-splenomegaly was not observed.

The children received regular blood transfusion treatment, with normal growth and development. Partial splenic embolization have been planned.

Blood routine showed moderate anemia, reticulocyte elevation. Bone marrow smear showed trilineage hyperplasia, erythroid hyperplasia was significantly active.

Health care department: The growth and development of the child were normal.

Blood routine: HB76 g/L, blood transfusion therapy

**March 8, 2017**

**June 4, 2017**

**September 20, 2017**

**February 21, 2018**

Second days after birth, the child was admitted to the Department of Newborn due to "skin yellowness 1 day "
